# Retrospective analysis of hemispheric structural network change as a function of location and size of glioma

**DOI:** 10.1093/braincomms/fcaa216

**Published:** 2020-12-17

**Authors:** Shawn D’Souza, Lisa Hirt, David R Ormond, John A Thompson

**Affiliations:** 1 MD Program, Virginia Commonwealth University, School of Medicine, Richmond, VA, USA; 2 Department of Neurosurgery, University of Colorado School of Medicine, Aurora, CO, USA; 3 Masters of Science in Modern Human Anatomy Program, University of Colorado School of Medicine, Aurora, CO, USA

**Keywords:** DTI, graph network, glioma, structural connectivity

## Abstract

Gliomas are neoplasms that arise from glial cell origin and represent the largest fraction of primary malignant brain tumours (77%). These highly infiltrative malignant cell clusters modify brain structure and function through expansion, invasion and intratumoral modification. Depending on the growth rate of the tumour, location and degree of expansion, functional reorganization may not lead to overt changes in behaviour despite significant cerebral adaptation. Studies in simulated lesion models and in patients with stroke reveal both local and distal functional disturbances, using measures of anatomical brain networks. Investigations over the last two decades have sought to use diffusion tensor imaging tractography data in the context of intracranial tumours to improve surgical planning, intraoperative functional localization, and post-operative interpretation of functional change. In this study, we used diffusion tensor imaging tractography to assess the impact of tumour location on the white matter structural network. To better understand how various lobe localized gliomas impact the topology underlying efficiency of information transfer between brain regions, we identified the major alterations in brain network connectivity patterns between the ipsilesional versus contralesional hemispheres in patients with gliomas localized to the frontal, parietal or temporal lobe. Results were indicative of altered network efficiency and the role of specific brain regions unique to different lobe localized gliomas. This work draws attention to connections and brain regions which have shared structural susceptibility in frontal, parietal and temporal lobe glioma cases. This study also provides a preliminary anatomical basis for understanding which affected white matter pathways may contribute to preoperative patient symptomology.

## Teaser

D’Souza *et al.* applied graph network measures to connectivity patterns in patients with unilateral glioma to assess global efficiency, modularity, and connectivity dependent on glioma localization, size, and distance from graph nodes. Results highlight cross-patient structural network changes that help identify susceptible hubs dependent on location of the lesion.

## Graphical Abstract

**Graphical Abstract:**
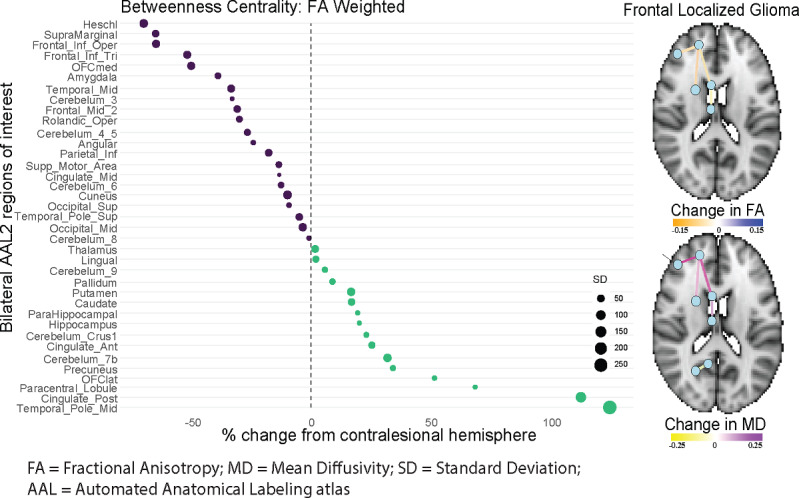

## Introduction

Gliomas are the most prevalent form of intrinsic brain tumour and are associated with several secondary effects on neural tissue including necrosis, breakdown of the blood–brain barrier and increased intracranial pressure (Raza *et al.*, 2002; Claes *et al.*, 2007; Goldbrunner *et al.*, 2018). Understanding *in vivo* glioma pathophysiology has relied on structural and functional magnetic resonance imaging (Maesawa *et al.*, 2015; Smits and van den Bent, 2017). As brain tumours invariably affect surrounding neural circuits, over the last two decades, diffusion tensor imaging (DTI), an *in vivo* method of extrapolating the white matter connections, has been used to study the global and targeted impact of tumour on white matter architecture and integrity (Assaf and Pasternak, 2008). In healthy subjects, DTI-based structural brain networks have been used to identify unique connectivity patterns that correlate with different cognitive functions and domains (Schmithorst and Wilke, 2002; Mayeli *et al.*, 2018; Zimmermann *et al.*, 2018). Similarly, DTI coefficients have been used as an indirect measure of white matter microstructural integrity, providing a proxy to *in vivo* quantification of axonal degeneration (Alexander *et al.*, 2007). Previous glioma-DTI studies have focused on targeted white matter bundles, such as the corticospinal tract and superior longitudinal fasciculus due to incidence in presenting patient symptomology and lesion proximity. However, the literature lacks a comprehensive structural connectivity analysis on lobe localized glioma cases.

Scientific application of graph network analysis provides a quantitative framework for understanding neural circuit connections. Through the application of graph theory, mathematical analyses on anatomical brain networks have allowed us to understand the interconnections between specific brain regions and the efficiency of information transfer at the local, hemispheric and global brain levels. When applied to the brain’s functional and structural interactions in both normal and diseased brain states, graph theory sheds light on how topology underlies function, new mechanisms of disease progression and may inform targets for neurosurgical intervention (Hart *et al.*, 2016; Farahani *et al.*, 2019). Of the pathological connectomes studied, few studies have analysed the extensive anatomical changes characteristic of glioma formation (Liu *et al.*, 2016).

In recent neuropathological studies, DTI structural connectivity analysis has highlighted anatomical changes in white matter circuitry to understand the functional changes and disease progression of non-glioma diseased brain states, such as Parkinson’s disease, schizophrenia and Alzheimer’s disease (Hagmann *et al.*, 2008). For example, the cognitive decline observed in Parkinson’s disease patients was found to be closely related to the decrease in nodal (local) efficiency, an indirect measure of the efficiency of local information transfer (Wang *et al.*, 2019). Similarly, a study by Leroux *et al.* (2020) on the tractographic alterations of patients with schizophrenia observed a link between the decreased structural integrity of the Superior Longitudinal Fasiculus (SLF) in the right hemisphere and impaired visuospatial attention. Additionally, structural and connectivity analysis of Alzheimer’s disease progression identified distinct tractographic changes characteristic of the various stages of the disease, including preclinical decline in connectivity predominantly in the temporal lobe, providing a potential method for early disease detection (Tucholka *et al.*, 2018).

Recent work applying network analyses to functional and structural connectomic data derived from gliomas have shown: (i) patterns of altered global connectivity depend on tumour grade in newly diagnosed glioma patients, (ii) lowered network efficiency correlates with cognitive performance in *IDH1* wildtype astrocytoma and (iii) preoperative connectometry improves the prognostic value of DTI in the context of high-grade gliomas (Liu *et al.*, 2016; Derks *et al.*, 2017; Kesler *et al.*, 2017). The goal of this current study is to better understand how lobe localized glioma uniquely affects the structural connectivity within the lesional hemisphere. Lesion affected nodes were identified by comparing network parameters between the lesional and non-lesional hemispheres, since in our study cohort, lesions were largely contained to one hemisphere. We measured the impact of glioma on node centrality [e.g. betweenness centrality (BC) and eigenvector centrality (EC)] and efficiency of information transfer [e.g. cluster coefficient (CC) and local efficiency (LE)] and identified connections between brain regions most vulnerable in the presence of various lobe localized gliomas extracted from the following DTI parameters: end-point fibre count, fractional anisotropy (FA) and mean diffusivity (MD). We hypothesize that lobe localization of glioma will exhibit a conserved pattern of highly affected nodes across the structural network measures.

## Methods

### Subject demographics

All procedures and protocols for this study were reviewed and approved by the Colorado Multi-Institutional Review Board (COMIRB 17-1136) and followed in accordance with the relevant guidelines and regulations. Subjects included in this study were patients undergoing resective surgery, from January 2016 to December 2017 at the University of Colorado Hospital, to remove an intracranial glioma. Though not a prerequisite for inclusion in this study, the DTI set available was classified by histopathology as glioma requiring functional imaging due to localization in or near language or motor cortex. Data were collected retrospectively from patient chart review through the application of a consent exempt institutional review board (IRB) protocol wherein only clinical data were reviewed after deidentification by a member of the study team. The initial subject population (*n* = 37) considered for the following novel analyses contained a subset of cases (*n* = 13) from two prior publications (Ormond *et al.*, 2017; D’Souza *et al.*, 2019). The initial patient population (*n* = 37) was then screened before analysis. Two cases were removed due to tumour infiltration of the contralateral hemisphere. One case was removed due to the tumour being located within the ventricle. The post-screening subject pool consisted of frontal lobe localized glioma cases (*n* = 22), parietal lobe localized glioma cases (*n* = 5), temporal lobe localized glioma cases (*n* = 6) and an occipital lobe localized glioma case (*n* = 1). The occipital lobe localized glioma case was discarded from the subject pool due to the small sample size. The post-screening subject pool consisted of 14 female patients (42%) and 19 male patients (58%) with an average age of 45.73 (standard deviation 16.51; range 20–80). Seven cases were histopathologically classified astrocytoma (21%), 18 as glioblastoma (GBM) (55%) and 8 as oligodendroglioma (24%). Nine cases were histopathologically classified as WHO Grade II (27%), 6 cases as WHO Grade III (18%) and 18 as WHO Grade IV (55%). Within the frontal lobe localized glioma group (*n* = 22), 13 patients were male (59%) and 9 patients were female (41%) with an average age of 44.64 (standard deviation 16.86; range 20–80). Within the parietal lobe localized group (*n* = 5), four patients were male (80%) and one patient was female (20%) with an average age of 55.60 (standard deviation 19.06; range 35–76). Within the temporal lobe localized group (*n* = 6), two patients were male (33%) and four patients were female (66%) with an average age of 41.50 (standard deviation 13.77; range 23–61). Each lobe localized subject pool consisted of a heterogeneous group of both glioma classification and grades (Table 1).

**Table 1:** **Subject demographics and registration to** Montreal Neurological Institute **space**

Case	Sex	Age	Side	Glioma pathology classification	Grade	Lobe	*R* ^2^
8	F	34	R	GBM	4	Frontal	0.7
20	M	56	R	GBM (epithelioid)	4	Frontal	0.75
22	M	22	L	GBM	4	Frontal	0.7
30	M	20	R	Oligodendroglioma	2	Frontal	0.76
31	M	54	R	GBM	4	Frontal	0.74
32	F	34	L	Oligodendroglioma	2	Frontal	0.78
36	M	30	R	GBM	4	Frontal	0.71
40	M	23	R	Astrocytoma	3	Frontal	0.7
41	F	58	R	GBM	4	Frontal	0.63
42	M	32	R	Astrocytoma	2	Frontal	0.66
43	M	35	L	Astrocytoma	2	Frontal	0.61
44	F	64	R	Oligodendroglioma	2	Frontal	0.69
48	F	36	R	GBM	4	Frontal	0.72
50	F	37	L	Oligodendroglioma	2	Frontal	0.74
51	F	48	L	Astrocytoma	3	Frontal	0.7
52	M	60	R	GBM	4	Frontal	0.72
53	M	35	L	Oligodendroglioma	3	Frontal	0.75
55	M	62	R	GBM	4	Frontal	0.71
57	M	80	R	GBM	4	Frontal	0.7
58	M	60	R	GBM	4	Frontal	0.61
59	F	34	L	Astrocytoma	2	Frontal	0.75
60	F	68	R	GBM	4	Frontal	0.66
11	M	38	R	Oligodendroglioma (Anaplastic)	3	Parietal	0.68
27	M	35	L	Oligodendroglioma (Anaplastic)	3	Parietal	0.71
38	F	73	L	GBM	4	Parietal	0.76
39	M	56	R	GBM	4	Parietal	0.64
54	M	76	R	GBM	4	Parietal	0.66
5	F	42	L	GBM	4	Temporal	0.69
35	F	23	R	Astrocytoma (diffuse)	2	Temporal	0.76
45	M	53	R	Astrocytoma	3	Temporal	0.7
46	F	61	R	GBM	4	Temporal	0.7
49	M	33	L	GBM	4	Temporal	0.73
56	F	37	L	Oligodendroglioma	2	Temporal	0.58

### Imaging sequence parameters

All images were obtained using a 3.0 T whole-body MR imager (Signa HDx; GE Medical Systems, Milwaukee, WI, USA) using single-shot echo-planar imaging. Acquisition times ∼9 min for DTI images. For DTI images, TE = 84.4 ms and TR = 16 000 ms with the diffusion gradient encoding in 32 directions at *b* = 1000 s/mm^2^ and an additional measurement without the diffusion gradient (*b* = 0 s/mm^2^). Data were recorded with a 128 × 128 spatial resolution in a 24 cm × 24 cm field of view. A total of 50 sections were obtained with a slice thickness of 2.6 mm and zero slice gap.

### Classification of lobe localization

Glioma cases were categorized under frontal, parietal or temporal depending on tumour location. Parameters were set to distinguish between tumours near the frontoparietal and temporoparietal borders. ITK Snap was utilized to manually segment the tumour volume from T2 MRI sequences (Yushkevich *et al.*, 2006). Next, the postcentral gyrus (fronto-parietal border) and superior temporal gyrus (temporo-parietal border) was identified for each case using the FreeSurfer-DKT Desikan-Killiany Atlas (Fischl *et al.*, 2004; Desikan *et al.*, 2006). If >51% of the tumour mass was anterior to the centroid of the postcentral gyrus ROI, the case was classified as frontal and if >51% of the tumour mass was posterior, it was classified as parietal. Similarly, if >51% of the tumour mass was superior to the centroid of the superior temporal gyrus, it was classified as parietal and if >51% of the tumour mass was inferior, the case was classified as temporal. Post-classification analysis found 75% of the tumours had >76% tumour volume in the designated lobe.

### Brain region parcellation

Each subject’s DTI image was linearly registered to the Montreal Neurological Institute 152 template through DSI Studio (*DSI Studio—A Tractography Software Tool*, n.d.). The results of each linear transformation (*R*^2^) between the subject’s DTI and the template's QA map is shown in Table 1 (range 0.58–0.78; mean 0.70; SD 0.05). Any *R*^2^ value lower than 0.6 was inspected manually for registration error (*n* = 1) as per DSI Studio Documentation and Tutorials ‘Create a Connectometry Database’ Tutorial (*Create a connectometry database - DSI Studio—A Tractography Software Tool*, n.d.). Following registration, brain parcellation was done using DSI Studio’s built-in automated anatomic labelling atlas 2 (AAL2) with 112 regions of interest (ROI) (56 per hemisphere and excluding vermis ROIs) (Rolls *et al.*, 2015). ROIs were transformed to each participant’s native space and were classified as nodes for the following connectometry analysis.

### White matter tract processing

All processing steps were conducted using DSI Studio. The diffusion data were reconstructed using q-space diffeomorphic reconstruction (Yeh and Tseng, 2011) to obtain the spin distribution function (Yeh *et al.*, 2010). A diffusion sampling length ratio of 1.25 was used. Restricted diffusion was quantified using restricted diffusion imaging (Yeh *et al.*, 2017). The b-table was checked by an automatic quality control routine to ensure its accuracy (Schilling *et al.*, 2019). Fibre tracking was conducted, based on whole-brain seeding (50 000 seeds), with a QA-assisted deterministic algorithm (Yeh *et al.*, 2013), using the following parameters, anisotropy threshold was automatically determined by DSI Studio following Montreal Neurological Institute 152 reconstruction, angular threshold of 45°, and step size of 0.5 voxels. Tracks with length shorter than 30 or longer than 300 mm were discarded. White matter tracts from whole-brain fibre tracking provided the edges needed for matrix computation and structural connectivity analysis.

### Computation of connectivity matrices

Weighted, undirected connectivity matrices for the frontal (*n* = 22), parietal (*n* = 5) and temporal (*n* = 6) cases were calculated in DSI Studio (*DSI Studio—A Tractography Software Tool*, n.d.). As described in the Brain Region Parcellation and White Matter Tract Processing sections, the AAL2 was utilized for ROI to node reconstruction and whole-brain tractography for tracts to edges respectively. Weighted, undirected connectivity matrices of the raw end-point tract count, end-point FA and end-point MD were generated for the contralesional (control) and ipsilesional hemispheres for all cases. Tracts that crossed into the opposing hemisphere were excluded to eliminate the confounding effect of the opposing hemisphere in subsequent analyses.

### Network analysis and data visualization

Group end-point tract count, FA and MD analyses were conducted using R Studio version 3.6.0. Steps for each analysis are described below. Graph network measures (listed below) were computed using the Brain Connectivity Toolbox (*Brain Connectivity Toolbox*, 2010; Rubinov and Sporns, 2010). Conversion of final significant increase/decrease end-point tract count, FA and MD data matrices into igraph arguments were achieved using R Studio version 3.6.0 igraph package (*CRAN - Package igraph*, 2020). Visualization of significant changes in brain connectivity and structural health on Montreal Neurological Institute space were generated using R Studio version 3.6.0 brainGraph package (*CRAN - Package brainGraph*, 2020).

### Graph network measures

The following four graph network measures were calculated for all ipsilesional and contralesional nodes, each weighted by either end-point tract count, FA or MD.

Cluster coefficient (CC): measure of cliquishness surrounding a node. The CC quantifies the fraction of how many neighbours of a node are also neighbours with each other. Arithmetically, the CC is the fraction of triangles surrounding node_i_ (Sporns and Kötter, 2004).

Local efficiency (LE): measure of how well node_i’_s neighbours are connected when node_i_ is removed from the network. Those nodes with lower LE indicate the node is crucial to the connectedness of surrounding nodes. Arithmetically, the LE is the inverse of the average shortest path length connecting all neighbours of node_i_ (Sporns and Kötter, 2004).

Betweenness centrality (BC): measure of the ‘gatekeeper’ characteristic of a node. Those nodes with a higher BC serve as a liaison between different cliques and are responsible for modulation of information influx. Arithmetically, the BC of node_i_ is the fraction of all shortest path lengths of a network that contain node_i_ (Sporns and Kötter, 2004).

Eigenvector centrality (EC): measure of how influential/‘high profile’ a node is. A node that has highly connected neighbours will have a higher EC (Sporns and Kötter, 2004).

### Hemispheric distribution analysis of graph network measures

The raw nodal CC, LE, BC and EC of the ipsilesional and contralesional hemisphere were log-normalized. Outlier data were identified and removed using a boxplot test. Anderson–Darling and Skewness-Kurtosis-Normality tests both classified the resulting distributions as non-normal (Anderson, 2011; Kim, 2013). Finally, a Wilcoxon 2 sample test, corrected for multiple comparisons using a false discovery rate, was run, comparing the distributions in raw nodal network measure of the ipsilesional versus contralesional hemispheres (Benjamini and Hochberg, 1995). An adjusted critical *P*-value of less than 0.05 was considered significant.

### Identification of highly impacted nodes based on glioma lobe localization

The percent change in end-point tract count, FA and MD weighted nodal network measure from the contralesional hemisphere was calculated for the entire subject pool (*n* = 33) using the following equation:

$$\%{\text{Change}}_{\text{Weighted Network Measure}}=\;\left(\left[{\text{Contralesional Node}}_{\text{Weighted Network Measure}}–{\text{ Analogous Ipsilesional Node}}_{\text{Weighted Network Measure}}\right]/{\text{Analogous Ipsilesional Node}}_{\text{Weighted Network Measure}}\right)\text{ x 1}00$$

This process was repeated after segregating the subject pool into its respective glioma lobe localization (frontal, parietal and temporal). The mean percent change across all weighted nodal network measures was calculated using the following equation:

Mean % change = [% change in end−point tract count + % change in FA + % change in MD]/3

Those nodes mean % change which fell in the top ten percentile for a given glioma lobar localization was identified (node_Top10_) and used for subsequent analyses.

### Lobar distribution analysis of graph network measures

Distribution plots of the mean percent change for each node_Top10_ in the presence of a lobe localized glioma was created for each network measure (CC, LE, BC, EC). A Kruskal–Wallis H-test compared the distributions of percent change in network measure from contralesional hemisphere based on glioma lobar localization. A *post hoc* Dunn’s test was run on those distributions which had an H-test *P*-value of <0.05. Significance was achieved for the *post hoc* Dunn’s test if *P*-values were <0.05.

### End-point tract count analysis

The mean end-point tract count values of the contralesional hemisphere for a given lobe localized glioma group was calculated and used to determine the 95% confidence interval. The corresponding mean end-point tract count values on the ipsilesional hemisphere were calculated. Those ipsilesional end-point tract counts which fell outside the 95% confidence interval were considered significantly different from contralesional end-point tract counts. The difference between significant mean ipsilesional end-point tract count and mean contralesional end-point tract count was calculated and plotted:

(Significant mean ipsilesional end-point tract count–mean contralesional end-point tract count=significant mean change in end-point tract count).

### FA and MD connectometry analysis

For FA and MD analyses, only those ipsilesional connections which were found to be significantly altered in end-point tract count were considered. The mean diffusion values of the contralesional hemisphere for a given lobe localized glioma group was calculated and used to find the 95% confidence interval. After calculating the corresponding mean diffusion values on the ipsilesional hemisphere, those values which fell outside the 95% confidence interval were defined as significant. The mean difference in diffusion value from the respective contralesional connections were plotted.

### Data availability statement

Network data, processing and analysis code are available from the corresponding author on reasonable request.

## Results

### Hemispheric changes in network measures

#### Cluster coefficient

**Figure 1:**
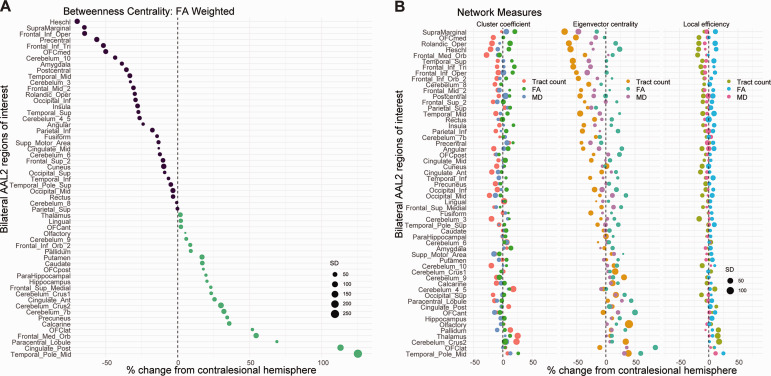
**Nodal percent change in network measure from contralesional hemisphere.** (**A**) Percent change in FA weighted BC from the contralesional hemisphere for each AAL2 identified node. (**B**) Compiled percent change dot plots of each network measure (CC, EC, LE) weighted by end-point tract count, FA, and MD for each AAL2 identified node. A list of all anatomical abbreviations is provided in Supplementary Table 1 (Rolls *et al.*, 2020).

For the entire subject population (*n* = 33, each dot point = 33 subjects × 2 hemispheres = 66), the distribution of weighted nodal CC of the ipsilesional and contralesional hemispheres were compared for statistical difference (weight of node: end-point tract count, FA, MD). With respect to end-point tract count weighted CC, the ipsilesional hemisphere showed a significant increase (*P*** **=** **1.21e−6). With respect to end-point FA weighted CC, the ipsilesional hemisphere showed a significant decrease (*P*** **=** **2.10e−5). With respect to end-point MD weighted CC, the ipsilesional hemisphere showed a significant increase (*P*** **=** **0.035). Figure 1B illustrates the percent change in Tract Count, FA and MD weighted CC from the contralesional hemisphere for each AAL2 node.

#### Local efficiency

For the entire subject population (*n* = 33, each dot point = 33 subjects × 2 hemispheres = 66), the distribution of weighted nodal LE of the ipsilesional and contralesional hemispheres were compared for statistical difference (weight of node: end-point tract count, FA, MD). With respect to end-point tract count weighted LE, the ipsilesional hemisphere showed a significant increase (*P*** **=** **9.60e−9). With respect to end-point FA weighted LE, the ipsilesional hemisphere showed a significant decrease (*P*** **=** **5.16e−12). With respect to end-point MD weighted LE, the ipsilesional hemisphere showed a significant increase (*P*** **=** **1.28e−5). Figure 1B illustrates the percent change in Tract Count, FA and MD weighted LE from the contralesional hemisphere for each AAL2 node.

#### Betweenness centrality

For the entire subject population (*n* = 33, each dot point = 33 subjects × 2 hemispheres = 66), the distribution of weighted nodal BC of the ipsilesional and contralesional hemispheres were compared for statistical difference (weight of node: end-point tract count, FA, MD). For end-point tract count, FA and MD weighted analysis, no differences were observed between the distribution of the ipsilesional hemisphere versus the contralesional hemisphere (*p*_BCCount_ = 0.31, *p*_BCFA_ = 0.16, *p*_BCMD_ = 0.095). Figure 1A and Supplementary Fig. 1 A and B illustrates the percent change in Tract Count, FA and MD weighted BC from the contralesional hemisphere for each AAL2 node.

#### Eigenvector centrality

For the entire subject population (*n* = 33, each dot point = 33 subjects × 2 hemispheres = 66), the distribution of weighted nodal EC of the ipsilesional and contralesional hemispheres were compared for statistical difference (weight of node: end-point tract count, FA, MD). With respect to end-point tract count weighted EC, the ipsilesional hemisphere showed a significant increase (*P*** **=** **1.76e−27). With respect to end-point FA weighted EC, the ipsilesional hemisphere showed no difference from the contralesional hemisphere (*P*** **=** **0.45). With respect to end-point MD weighted EC, the ipsilesional hemisphere showed a significant increase (*P*** **=** **1.95e−8). Figure 1B illustrates the percent change in Tract Count, FA and MD weighted EC from the contralesional hemisphere for each AAL2 node.

### Volumetric and distance analysis

A Pearson correlation test was conducted (*n* = 33) comparing the tumour volume to percent change from the contralesional hemisphere across all DTI-connectivity measures. tumour volume was not correlated to percent change (*r* = 0.054; *P* = 0.77). A subsequent tumour volume correlation was conducted, segregating the subject pool based on tumour lobe location with results showing no significant correlation across all three locations (Frontal: *r* = 0.12, *P* = 0.6; Parietal: *r* = −0.42, *P* = 0.48; Temporal: *r* = 0.24, *P* = 0.65).

**Figure 2:**
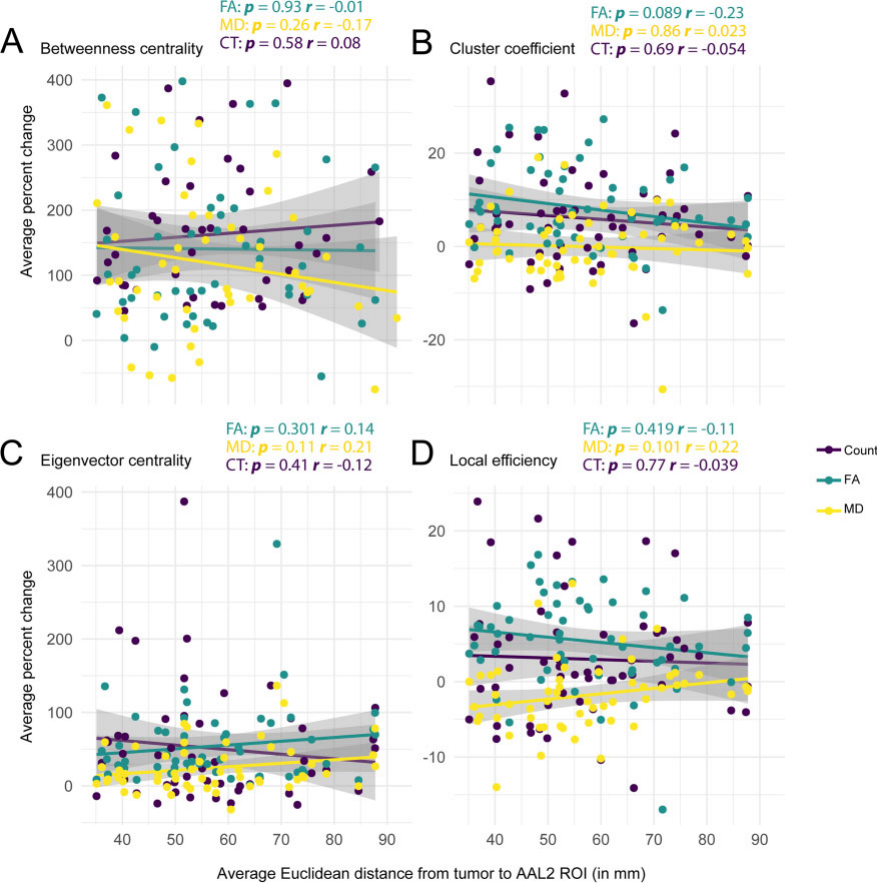
**Euclidian distance from tumour (mm) versus average percent change in DTI-connectivity measure.** (**A**) Correlation analysis between the average Euclidian distance of ipsilesional AAL2 node from centroid of tumour ROI (mm) against the average percent change in BC count, FA and MD from the corresponding contralesional AAL2 node. (**B**) Correlation analysis between the average Euclidian distance of ipsilesional AAL2 node from centroid of tumour ROI (mm) against the average percent change in CC count, FA, and MD from the corresponding contralesional AAL2 node. (**C**) Correlation analysis between the average Euclidian distance of ipsilesional AAL2 node from centroid of tumour ROI (mm) against the average percent change in EC count, FA, and MD from the corresponding contralesional AAL2 node. (**D**) Correlation analysis between the average Euclidian distance of ipsilesional AAL2 node from centroid of tumour ROI (mm) against the average percent change in LE count, FA and MD from the corresponding contralesional AAL2 node.

A distance correlation was also conducted (each dot point = 33 subjects × 2 hemispheres × 56 AAL2 brain regions) comparing the DTI-Connectivity measure from the non-tumour hemisphere at each AAL2 ROI to the average Euclidean distance from the tumour to the AAL2 ROI (Fig. 2). No significant correlations were noted (BC: FA: *r* = −0.01, *P* = 0.93; MD: *r* = −0.17, *P* = 0.26; Count [CT]: *r* = 0.08, *P* = 0.58) (CC: FA: *r* = −0.23, *P* = 0.089; MD: *r* = 0.023, *P* = 0.86; Count [CT]: *r* = −0.054, *P* = 0.69) (EC: FA: *r* = 0.14, *P* = 0.301; MD: *r* = 0.21, *P* = 0.11; Count [CT]: *r* = −0.12, *P* = 0.41) (LE: FA: *r* = −0.11, *P* = 0.419; MD: *r* = 0.22, *P* = 0.101; Count [CT]: *r* = −0.039, *P* = 0.77).

### Localized changes in network measures

Table 2 reports the following nodes which were in the top 10 percentile of mean percent change in frontal (*n* = 22), parietal (*n* = 5) and temporal (*n* = 6) lobe localized glioma cases (Mean % change = [% change in count + % change in FA + % change in MD]/3). The mean percent change for each node was plotted for all network measures (CC, LE, BC, EC) in the presence of frontal, parietal and temporal lobe localized gliomas.

**Table 2:** Nodes in the top ten percentile of mean percent change from contralesional hemisphere versus glioma lobe localization

Tumour location	Frontal				Parietal				Temporal			
Net measure	CC	LE	BC	EC	CC	LE	BC	EC	CC	LE	BC	EC
Top ten percentile frontal nodes												
Occipital_mid	−18.12 ± −27.92	−2.42 ± −15.22	52.43 ± −6.39	34.86 ± −13.25	−25.27 ± −69.66	−7.37 ± −31.07	−46.88 ± −77.31	−15.05 ± −39.79	22.29 ± 10.19	5.07 ± 0.63	−15.70 ± −22.09	−33.87 ± −31.28
Occipital_Sup	−6.09 ± 0.75	−1.78 ± −8.66	6.48 ± 28.87	31.55 ± 28.38	−16.34 ± 40.20	−12.11 ± −4.62	245.65 ± 451.39	−7.05 ± 43.47	11.52 ± 36.93	2.96 ± 3.18	−46.19 ± −64.44	5.42 ± 0.32
OFClat	6.81 ± −10.95	17.32 ± −3.27	345.06 ± 135.56	73.67 ± 31.60	38.39 ± 35.66	27.70 ± 23.78	−72.97 ± −73.77	25.45 ± −6.60	−27.51 ± −6.70	−24.41 ± 3.55	19.87 ± −35.42	−4.06 ± 10.08
Olfactory	20.00 ± 10.62	14.62 ± 13.14	−19.46 ± −40.48	31.88 ± 42.11	−34.09 ± −40.56	−21.65 ± 12.51	1115.37 ± 1267.01	24.02 ± −5.02	−23.45 ± 7.36	−21.20 ± 81.60	−20.10 ± 39.02	−22.11 ± 39.25
Rolandic_Oper	−3.65 ± −18.53	−5.19 ± −12.56	10.72 ± 71.05	−24.82 ± 6.63	−18.93 ± −25.24	−6.85 ± −2.45	101.85 ± 75.35	−12.10 ± −14.13	16.03 ± −47.16	6.07 ± −43.45	−63.43 ± −66.91	−26.15 ± −53.62
SupraMarginal	4.75 ± −9.92	−2.63 ± −12.29	−62.03 ± −63.20	−46.00 ± −58.33	4.92 ± 44.55	3.00 ± 40.24	−64.49 ± −51.46	−34.04 ± −41.04	32.07 ± 62.72	13.58 ± 19.58	−50.27 ± −33.01	−44.54 ± −51.87
Top ten percentile parietal nodes												
Cerebelum_3	11.69 ± 2.32	1.92 ± −5.42	−58.47 ± −39.86	0.54 ± 0.16	−36.72 ± −43.17	−28.13 ± 111.47	NA	73.37 ± 33.97	7.21 ± −30.03	6.81 ± −59.19	53.30 ± 60.01	−7.05 ± −28.99
Cerebelum_4_5	8.80 ± 2.13	2.51 ± 12.35	−27.94 ± −33.48	−8.80 ± −5.77	6.87 ± 78.08	2.01 ± 29.86	62.40 ± 64.90	64.42 ± 210.85	33.51 ± 7.47	13.34 ± 9.01	−71.90 ± −75.89	−14.07 ± 18.06
OFCant	−16.22 ± −28.21	2.86 ± −7.37	47.30 ± 19.87	24.11 ± 37.76	−17.10 ± −4.42	1.77 ± −15.98	−13.11 ± −42.47	53.04 ± 128.42	23.09 ± −38.54	20.61 ± −47.08	6.00 ± −16.03	−1.65 ± −8.23
OFClat	6.81 ± −10.95	17.32 ± −3.27	345.06 ± 135.56	73.67 ± 31.60	38.39 ± 35.66	27.70 ± 23.78	−72.97 ± −73.77	25.45 ± −6.60	−27.51 ± −6.70	−24.41 ± 3.55	19.87 ± −35.42	−4.06 ± 10.08
Parietal_Sup	7.61 ± 26.34	0.30 ± 9.11	−0.59 ± −9.02	−15.32 ± −5.45	2.20 ± 1.02	1.08 ± 36.92	−78.40 ± −81.58	−56.12 ± −71.63	17.82 ± −10.98	2.16 ± −29.16	−34.22 ± −13.33	−29.00 ± 6.60
Temporal_Pole_Mid	33.18 ± −6.24	21.74 ± −14.49	−15.92 ± −1.49	8.47 ± 4.27	−8.69 ± −10.63	−6.70 ± −11.58	NA	38.82 ± 51.89	−16.22 ± −41.89	16.15 ± −29.85	946.16 ± 623.93	421.77 ± 481.63
Top ten percentile temporal nodes												
Frontal_Med_Orb	−19.43 ± −25.07	−12.58 ± 36.31	12.50 ± 27.16	−14.80 ± 1.81	21.28 ± −58.54	10.45 ± −62.51	75.40 ± 27.68	−8.61 ± −63.29	−16.80 ± −33.61	−2.06 ± −22.53	−4.85 ± −23.33	56.83 ± 125.94
OFCpost	−6.78 ± −3.15	1.67 ± 15.03	−19.59 ± −16.33	−0.48 ± 4.77	−13.80 ± 40.87	−8.29 ± 93.07	56.31 ± 64.75	−15.47 ± −2.43	−14.55 ± −23.52	−3.98 ± −18.80	15.61 ± −11.36	48.24 ± 241.01
Paracentral_Lobule	−4.68 ± 26.61	−0.43 ± 2.31	108.97 ± 94.32	15.84 ± −3.12	−8.41 ± −30.65	0.09 ± 2.99	0.15 ± −68.92	0.32 ± −9.32	−17.60 ± −13.33	11.25 ± 37.45	315.82 ± 131.08	96.11 ± 32.70
Supp_Motor_Area	−13.41 ± −41.86	−2.37 ± −7.95	30.59 ± 52.03	11.54 ± 2.04	−3.00 ± −16.38	6.35 ± −7.66	−12.02 ± −22.31	17.63 ± −26.01	−13.23 ± −31.99	6.01 ± −0.95	105.26 ± 37.67	47.44 ± 15.67
Temporal_Pole_Mid	33.18 ± −6.24	21.74 ± −14.49	−15.92 ± −1.49	8.47 ± 4.27	−8.69 ± −10.63	−6.70 ± −11.58	NA	38.82 ± 51.89	−16.22 ± −41.89	16.15 ± −29.85	946.16 ± 623.93	421.77 ± 481.63
Temporal_Pole_Sup	−0.88 ± −3.52	1.65 ± −20.08	−12.39 ± −25.16	−0.10 ± −24.31	4.77 ± 8.42	21.54 ± −16.47	−55.87 ± −79.77	−28.07 ± −51.42	−19.28 ± −21.09	−4.30 ± −10.69	310.67 ± 264.04	52.24 ± 47.14

Mean % change from contralesional hemisphere (Count, FA, MD).

A list of all anatomical abbreviations is provided in Supplementary Table 1.

**Figure 3:**
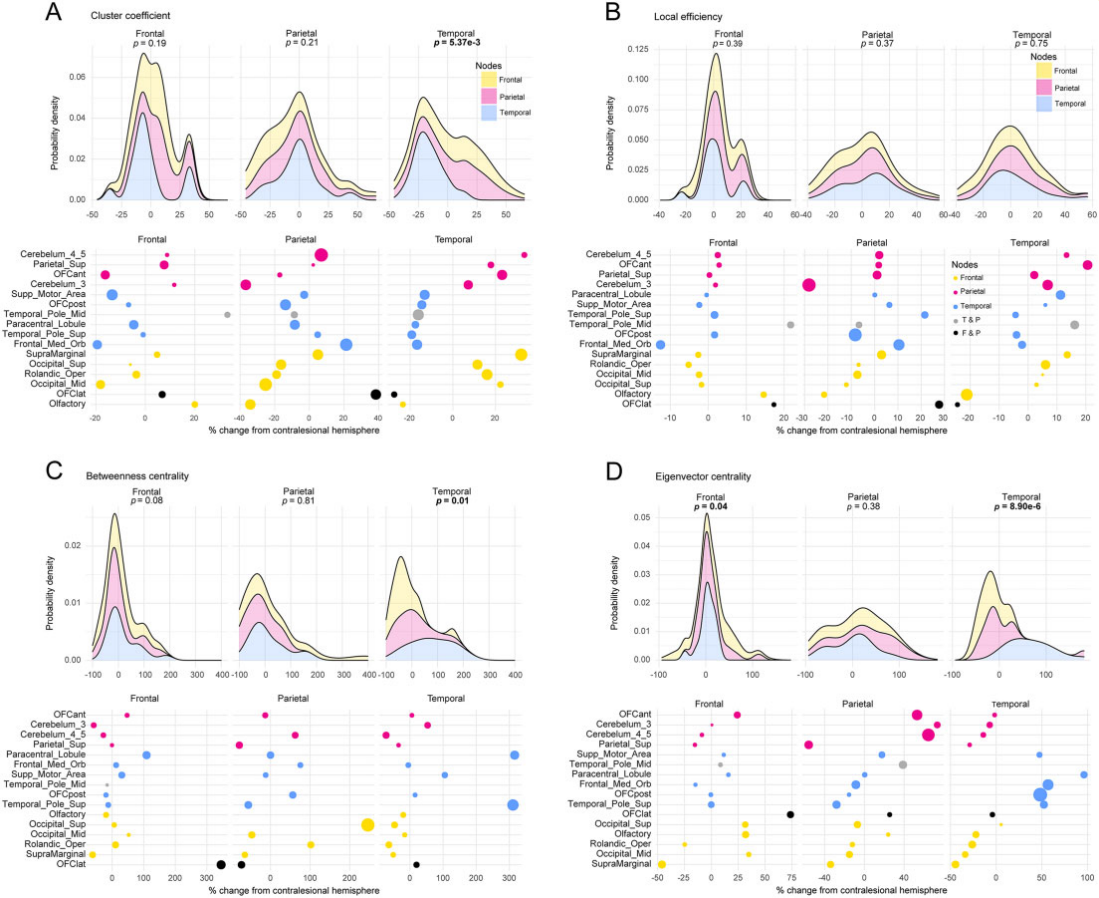
**Distribution of mean percent change in top 10 percentile AAL2 brain regions affected by glioma lobe localization.** (**A**) Density and dot plot visualization of the percent change in CC of the top 10 percentile AAL2 nodes affected by glioma lobe localization. H-test revealed the distribution of percent change in CC in these nodes was found to be significantly different in temporal lobe localized gliomas. (**B**) Density and dot plot visualization of the percent change in LE of the top 10 percentile AAL2 nodes affected by glioma lobe localization. H-test revealed no significantly different distributions between the three lobe localized glioma groups. (**C**) Density and dot plot visualization of the percent change in BC of the top 10 percentile AAL2 nodes affected by glioma lobe localization. H-test revealed the distribution of percent change in BC in these nodes was found to be significantly different in temporal lobe localized gliomas. (**D**) Density and dot plot visualization of the percent change in EC of the top 10 percentile AAL2 nodes affected by glioma lobe localization. H-test revealed the distribution of percent change in EC in these nodes was found to be significantly different in frontal and temporal lobe localized gliomas. A list of all anatomical abbreviations is provided in Supplementary Table 1 (Rolls *et al.*, 2020).

Figure 3A–D shows the distribution of the 33 subjects × 2 hemispheres. With respect to CC (Fig. 3A), the difference in distributions of mean percent change for all frontal, parietal and temporal affected nodes was not significant in the presence of a frontal (*P* = 0.19) or parietal (*P* = 0.21) lobe localized glioma. The distributions were significantly different in the presence of a temporal lobe localized glioma (*P* = 5.31e−3). *Post hoc* Dunn’s test on distributions of temporal lobe localized gliomas revealed a significant difference in mean percent change distribution between frontal affected nodes compared to temporal affected nodes (*P* = 0.02) and parietal affected nodes compared to temporal affected nodes (*P* = 7.60e−3), and no significant difference in mean percent change distribution between frontal affected nodes compared to parietal affected nodes (*P* = 0.61).

With respect to LE (Fig. 3B), the difference in distributions of mean percent change for all frontal, parietal and temporal affected nodes was not significant in the presence of a frontal (*P* = 0.39), parietal (*P* = 0.37) or temporal (*P* = 0.75) lobe localized glioma.

With respect to BC (Fig. 3C), the difference in distributions of mean percent change for all frontal, parietal, and temporal affected nodes was not significant in the presence of a frontal (*P* = 0.08) or parietal (*P* = 0.81) lobe localized glioma. The distributions were significantly different in the presence of a temporal lobe localized glioma (*P* = 0.01). *Post hoc* Dunn’s test on distributions of temporal lobe localized gliomas revealed a significant difference in mean percent change distribution between frontal affected nodes compared to temporal affected nodes (*P* = 0.02), and no significant difference in mean percent change distribution between frontal affected nodes compared to parietal affected nodes (*P* = 0.60) and parietal affected nodes compared to temporal affected nodes (*P* = 5.51e−2).

With respect to EC (Fig. 3D), the difference in distributions of mean percent change for all frontal, parietal and temporal affected nodes was not significant in the presence of a parietal lobe localized glioma (*P* = 0.38). The distributions were significantly different in the presence of a frontal (*P* = 0.04) and temporal (*P* = 8.90e−6) lobe localized glioma. *Post hoc* Dunn’s test on distributions of frontal lobe localized gliomas revealed a significant difference in mean percent change distribution between frontal affected nodes compared to temporal affected nodes (*P* = 0.04), and no significant difference in mean percent change distribution between frontal affected nodes compared to parietal affected nodes (*P* = 0.33) and parietal affected nodes compared to temporal affected nodes (*P* = 0.33). *Post hoc* Dunn’s test on distributions of temporal lobe localized gliomas revealed a significant difference in mean percent change distribution between frontal affected nodes compared to temporal affected nodes (*P* = 4.01e−5) and parietal affected nodes compared to temporal affected nodes (*P* = 1.42e−4), and no significant difference in mean percent change distribution between frontal affected nodes compared to parietal affected nodes (*P* = 0.70).

### Connectivity changes in the presence of various lobe localized gliomas

#### Differences in end-point tract count

Ipsilesional connections which exhibited a significant difference in end-point tract count from the contralesional hemisphere were divided into two groups: increased and decreased end-point tract count. Table 3 (Frontal), 4 (Parietal) and 5 (Temporal) lists the significantly increased and decreased ipsilesional connections and the corresponding mean increase/decrease from the contralesional hemisphere. With respect to AAL2.120 atlas anatomy, these results were plotted in Montreal Neurological Institute space (Supplementary Fig. 2). The left hemisphere was arbitrarily designated the ipsilesional hemisphere.

**Table 3:** Frontal lobe localized glioma: end point, FA and MD analysis summary

Node A	Node B	Mean increase in end-point count	Contralesional FA	Ipsilesional FA	Contralesional MD	Ipsilesional MD
SFG.L	IFGtriang.L	18.18	0.38^a^	0.33^a^	0.88^b^	1.10^b^
SFG.L	SMA.L	11.41	0.39^a^	0.33^a^	0.94^b^	1.13^b^
SMA.L	MCC.L	10.05	0.37^a^	0.34^a^	0.93^b^	1.01^b^
STG.L	MTG.L	10.27				
CB8.L	CB9.L	2.73			0.90^a^	0.83^a^

Node A	Node B	Mean decrease in end-point count	Contralesional FA	Ipsilesional FA	Contralesional MD	Ipsilesional MD
SFG.L	PUT.L	22.50	0.40^a^	0.36^a^	0.86^b^	0.95^b^

a Significant decrease.

b Significant increase.

A list of all anatomical abbreviations is provided in Supplementary Table 1.

Frontal: With respect to the ipsilesional hemisphere, we observed a higher number of connections which exhibited an increase in end-point tract count compared to decreased end-point tract count (ratio_increased: decreased_: 5:1). With respect to the nodes of connections which showed an increase in end-point tract count, 37% (3/8) were located in the frontal lobe, 25% (2/8) were located in the temporal lobe, 25% (2/8) were located in the cerebellum and 13% (1/8) were located in the limbic system. For the one connection which showed a decrease in ipsilesional end-point tract count, one of the nodes was present in the frontal lobe (50%; 1/2) and one was located in subcortical gray matter (50%; 1/2).

Parietal: With respect to the ipsilesional hemisphere, we observed a higher number of connections which exhibited an increase in end-point tract count compared to decreased end-point tract count (ratio_increased: decreased_: 9:2). With respect to the nodes of connections which showed an increase in end-point tract count, 16% (2/12) were located in the parietal lobe, 16% (2/12) were located in the temporal lobe, 16% (2/12) were located in the frontal lobe, 41% (5/12) were located in the occipital lobe and 8% (1/12) were located in the limbic system. With respect to the nodes of connections which showed a decrease in end-point tract count, 50% (2/4) of the nodes were located in the frontal lobe and 50% (2/4) were located in the occipital lobe.

Temporal: No connections were found to have had a significant decrease in tract count from the contralesional hemisphere. Again, we observed a higher number of connections which exhibited an increase in end-point tract count compared to decreased end-point tract count (ratio_increased: decreased_: 15:0). With respect to the nodes of connections which showed an increase in end-point tract count, 14% (3/21) were located in the temporal lobe, 10% (2/21) were located centrally, 14% (3/21) were located in the limbic system, 5% (1/21) were located in the subcortical grey matter, 33% (7/21) were located in the occipital lobe, 10% (2/21) were located in the parietal lobe and 14% (3/21) were located in the frontal lobe.

#### FA analysis

**Figure 4:**
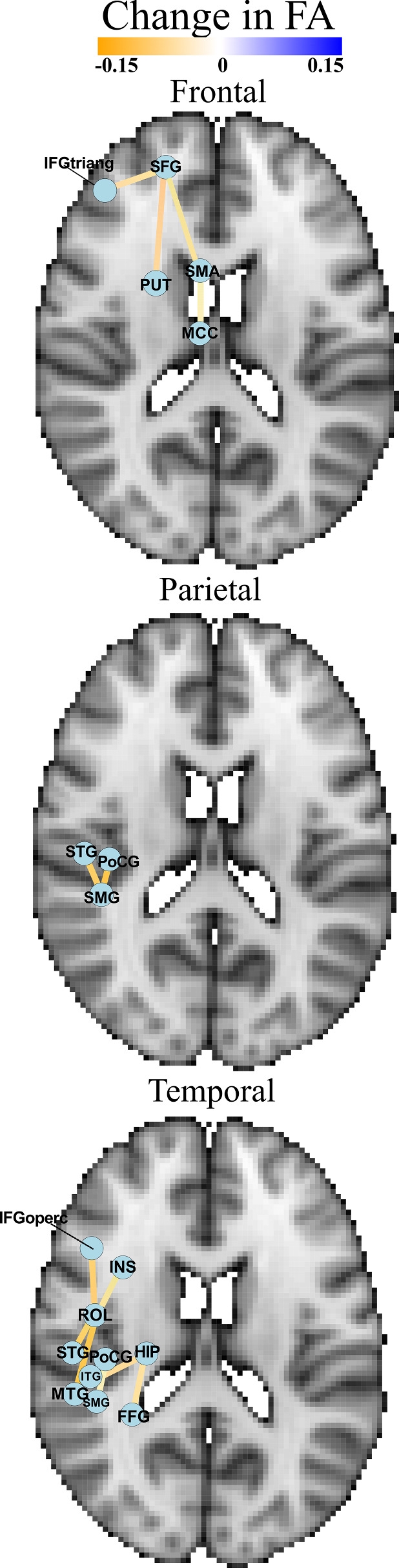
**Mean change in FA in significantly altered connections of frontal, parietal and temporal lobe localized glioma cases.** An axial anatomical representation of mean change in FA of significantly affected connections (by change in end-point tract count) in frontal, parietal, and temporal lobe localized glioma cases. A list of all anatomical abbreviations is provided in Supplementary Table 1 (Rolls *et al.*, 2020).

The FA was calculated for all significantly increased/decreased ipsilesional connections. Tables 3, 4 and 5 highlight those connections which exhibited both a significant change in count and significant decrease in FA from their contralesional counterparts. Figure 4 anatomically plots the mean change in FA of these connections on the left hemisphere (arbitrarily chosen to represent the ipsilesional hemisphere).

**Table 4:** Parietal lobe localized glioma: end point count, FA and MD analysis summary

Node A	Node B	Mean increase in end-point count	Contralesional FA	Ipsilesional FA	Contralesional MD	Ipsilesional MD
MFG.L	SFGmedial.L	12.6				
CAL.L	LING.L	5				
CAL.L	SOG.L	1.8				
CUN.L	SOG.L	4.8				
CUN.L	MOG.L	6.4				
PoCG.L	SMG.L	17.2	0.35^a^	0.24^a^	0.89^b^	1.05^b^
INS.L	STG.L	17.4				
INS.L	MTG.L	12.4				
SMG.L	STG.L	11	0.33^a^	0.24^a^		

Node A	Node B	Mean decrease in end-point count	Contralesional FA	Ipsilesional FA	Contralesional MD	Ipsilesional MD
SFG.L	SMA.L	4.4				
LING.L	IOG.L	6				

a Significant decrease.

b Significant increase.

A list of all anatomical abbreviations is provided in Supplementary Table 1.

**Table 5:** Temporal lobe localized glioma: end-point count, FA and MD analysis summary

Node A	Node B	Mean increase in end-point count	Contralesional FA	Ipsilesional FA	Contralesional MD	Ipsilesional MD
IFGoperc.L	ROL.L	6	0.36^a^	0.32^a^	0.89^b^	0.98^b^
IFGtriang.L	INS.L	11			0.91^b^	1.00^b^
ROL.L	INS.L	17.83	0.33^a^	0.28^a^	0.93^b^	1.08^b^
SMA.L	MCC.L	23.17				
CAL.L	LING.L	7.17				
CUN.L	SOG.L	4.83				
LING.L	IOG.L	4.17				
MOG.L	IOG.L	8.5				
HIP.L	FFG.L	17.17	0.34^a^	0.29^a^		
PoCG.L	SPG.L	11.33				
PoCG.L	SMG.L	12.17	0.36^a^	0.33^a^	0.84^b^	0.89^b^
INS.L	PUT.L	11.83				
ROL.L	STG.L	11	0.35^a^	0.25^a^	0.89^b^	1.12^b^
ROL.L	MTG.L	13.17	0.41^a^	0.30^a^	0.83^b^	1.03^b^
HIP.L	ITG.L	4.67	0.38^a^	0.31^a^		

a Significant decrease.

b Significant increase.

A list of all anatomical abbreviations is provided in Supplementary Table 1.

Frontal: Of these connections, 60% (3/5) of the nodes were located in the frontal lobe, 20% (1/5) were located in the subcortical grey matter and 20% (1/5) were located in the limbic system. No connections were identified to have had both a significant change in count and increase in FA.

Parietal: Of these connections, 66% (2/3) of nodes were located in the parietal lobe and 33% (1/3) were located in the temporal lobe. No connections were identified to have had both a significant change in count and increase in FA.

Temporal: Of these connections, 30% (3/10) of the nodes were located in the temporal lobe, 20% (2/10) were located centrally, 20% (2/10) were located in the limbic system, 10% (1/10) were located in the frontal lobe, 10% (1/10) were located in the parietal lobe and 10% (1/10) were located in the occipital lobe. No connections were identified to have had both a significant change in count and an increase in FA.

#### MD analysis

The MD was calculated for all significantly increased/decreased ipsilesional connections. Tables 3, 4 and 5 highlight those connections which exhibited both a significant change in count and significant increase or decrease in MD from their contralesional counterparts. Supplementary Figure 3 anatomically plots the mean change in MD of these connections on the left hemisphere (arbitrarily chosen to represent the ipsilesional hemisphere).

Frontal: Of these connections, those which showed a significant increase in MD, 60% (3/5) of the nodes were located in the frontal lobe, 20% (1/5) were located in the subcortical grey matter and 20% (1/5) were located in the limbic system. Of the one connection which showed a decrease in MD, both nodes (2/2) were located in the cerebellum.

Parietal: Of these connections, all nodes (2/2) were located in the parietal lobe. No connections were identified to have had both a significant change in count and decrease in MD.

Temporal: Of these connections, 25% (2/8) of the nodes were located in the temporal lobe, 13% (1/8) were located in the limbic system, 25% (2/8) were located centrally, 25% (2/8) were located in the frontal lobe, and 13% (1/8) were located in the parietal lobe. No connections were identified to have had both a significant change in count and decrease in MD.

## Discussion

Prior simulation studies testing the resiliency of the brain’s small-world connectivity in light of targeted attacks indicated a fitness value that mitigated the loss of network functionality in the presence of pathology (Achard *et al.*, 2006). This study further explores the brain’s response to targeted attacks by assessing the changes in structural brain network properties at the hemispheric and focal levels in the presence of various lobe localized gliomas. Results were indicative of elevated ipsilesional connectedness with respect to integration measures (CC, LE) and modularity measures (EC); however, the structural integrity of these connections were diminished compared to the contralesional hemisphere. The network property changes observed in the ipsilesional hemisphere were attributed to a unique set of AAL2 anatomical regions depending on glioma lobe localization. Finally, ipsilesional connectivity network analysis on raw end-point tract count, FA and MD identified connections most susceptible in either frontal, parietal or temporal localized glioma cases. These findings were consistent with previous works on white matter structural health and our hypothesis stating conserved patterns of nodal susceptibility based on glioma localization.

This study reflects and extends previous works through the observation of increased levels of ipsilesional integration and modularity. Consistent with previous research that has shown an increase in ipsilesional raw fibre count (Ormond *et al.*, 2017), in this study, we observed that count weighted hemispheric network analysis showed increased levels of ipsilesional CC and LC, measures of cliquishness and efficiency of local information flow respectively (Vecchio *et al.*, 2017). In addition, an increase in ipsilesional EC, a modulatory measure correlated to the ‘high-profileness’ of a node, was observed (Vecchio *et al.*, 2017). Similar patterns of increased integration within the ipsilesional hemisphere have been reported in cerebral glioma cases, with one study finding increased functional integration of the hippocampus (Esposito *et al.*, 2012). However, from a structural connectivity standpoint, though consistent with previous findings of increased ipsilesional count, these results are inconsistent with the anatomical effects of glioma presence, which include tract displacement (Schonberg *et al.*, 2006; Angeli *et al.*, 2018). With respect to the structural integrity of integration and modularity measures, we observed an ipsilesional decline. FA and MD weighted hemispheric network analysis showed decreased levels of structural integrity (decreased FA, increased MD) with respect to CC cliquishness, LE local information integration and EC ‘high-profileness’. Structural integrity findings were consistent with both previous studies and glioma pathophysiology, particularly GBM, microstructural symptoms which include necrosis and degradation of surrounding neural tissue (D’Alessio *et al.*, 2019). We hypothesize our new findings could be due to cortical neuroplastic reorganization of the ipsilesional hemisphere in an attempt to preserve cognitive function during the early stages of tumour growth (Kong *et al.*, 2016).

Previous studies have shown the localized effect of gliomas on surrounding microstructural integrity of white matter fibres, noting an increase in degradation with increasing proximity (D’Souza *et al.*, 2019). However, the widespread, non-microstrucutral changes have yet to be defined (Fig. 1A and B, Supplementary Fig. 1 A and B). In accordance with our hypothesis, each lobe localized glioma had unique sets of nodes which contributed to the observed hemispheric changes in network measure (Table 2). However, of the nodes most affected, 2/6 most affected nodes were located in the frontal lobe in frontal glioma cases, 1/6 located in the parietal lobe in parietal cases and 2/6 in the temporal lobe in temporal cases. All other nodes were located at a distal point with respect to the lesional lobe. Not only do these results specify the brain regions most likely to exhibit a change in network characteristic in the presence of various lobe localized gliomas but also indicate changes in brain connectivity occurring distally from the point of lesion. Similar cortical reorganization has been observed in glioma cases undergoing a second resection. A study done by Southwell *et al.* (2016) showed functional regions within the tumour parenchyma during the initial resection using direct electrical stimulation (DES) (Southwell *et al.*, 2016). However, these same areas were no longer functional during the second resection. These data also point to a cortical neuroplastic mechanism taking place during the growth of lesion, some data also suggesting distal change as far as the recruitment of the contralesional hemisphere to preserve function (Kong *et al.*, 2016; Schlemm *et al.*, 2020).

To further explore the effects of tumour volume and nodal distance from tumour on the percent change in DTI-connectivity measure, volume and distance (Fig. 2A–D) correlations were conducted. The volume analysis showed no significance, suggesting lesion volume, which was further grouped by lobe location, did not influence the connectivity changes observed. Similarly, the distance correlation was insignificant, suggesting distance did not influence the changes observed in the previous and following analyses, but rather lobe localization.

The subsequent distribution analysis allowed us to determine which network measures were most affected for each node_Top10_ (Fig. 3A–D). For node_Top10Temporal_, cliquishness (CC), gate-keeper effect (BC) and high-profileness (EC) were highly affected in the presence of a temporal lobe lesion compared to node_Top10Frontal_ (CC, BC, EC) and node_Top10Parietal_ (CC, EC). For node_Top10Frontal_, high-profileness (EC) was highly affected in the presence of a frontal lobe lesion compared to node_Top10Temporal_. Node_Top10Parietal_ did not have a specific measure which was highly affected given a parietal lobe lesion compared to node_Top10Temporal and_ node_Top10Frontal_. In other words, given a frontal lobe localized glioma, node_Top10Frontal_ were more susceptible to changes in EC, given a parietal lobe localized glioma, node_Top10Parietal_ was no more susceptible to a change in any network property measure, and given a temporal lobe localized glioma, node_Top10Temporal_ was more susceptible to changes in CC, BC and EC. Again, this observed change in network property could be attributed to cortical neuroplasticity. It is hypothesized that higher order cortical areas (high EC) have lower plasticity potential versus regions that are more involved in widespread transmission (high BC) (Kong *et al.*, 2016). The network property changes seen in these nodes may be due to a higher potential for plasticity; however, further graph theory network studies would need to be conducted on non-diseased brain states to better understand the respective network properties of the node_Top10_.

Finally, structural connectivity analysis was conducted to identify specific pathways which exhibited a count change and the underlying integrity of those connections in the presence of various lobe localized gliomas (Fig. 4, Supplementary Figs 2 and 3). Several affected connections had at least one node proximal to the lesion location. Most connections also showed an increase in fibre count while also exhibiting a decrease in structural integrity. This is consistent with previous works stating tumour impact on microstructural integrity is directly correlated to lesion proximity (Mair *et al.*, 2018; D’Souza *et al.*, 2019). An increase in connections between several nodes, though consistent with previous findings of increased ipsilesional fibre count, warrants further exploration (Kong *et al.*, 2016; Ormond *et al.*, 2017). One possible explanation attributes this to the cortical neuroplasticity described earlier, primarily the idea of recruitment of contralesional brain regions to aid its ipsilesional counterparts, a phenomenon noted in functional recovery of stroke patients (Small *et al.*, 2002). Another could be mass effect concentrating surrounding fibres, providing the fibre tracking algorithm to read an increase in connectivity between two lesion proximal nodes (Schonberg *et al.*, 2006; Angeli *et al.*, 2018). Finally, the presence of oedema could be affecting fibre tracking, feigning the presence of increased connectivity while also showing an decrease in FA and increase mean diffusivity (Field and Alexander, 2004; Wu *et al.*, 2004; Bulakbaşı, 2009).

### Limitations and future directions

This study provided preliminary data highlighting the network property and structural connectivity changes present in frontal, parietal and temporal lobe localized glioma cases. However, this study had limitations which should be addressed in future studies. The small sample size for both parietal (*n* = 5) and temporal lobe cases (*n* = 6) makes these data susceptible to both outliers and false negatives. The second limitation of this study was the absence of histological confirmation of tumour impact on white matter integrity for each case, specifically in the anatomical areas highlighted to have shown decreased integrity. This would have helped confirm the results of the FA and MD analyses by giving physical observable evidence of white matter degradation. Furthermore, correlation of structural connectivity changes to clinical functional presentation should be addressed in future studies to begin understanding if neuroplastic compensation is occurring. In addition, since this study used a within-subject experimental design to compare connectivity changes between the ipsilesional and contralesional hemispheres, we included only those subjects with unilaterally localized glioma, which limits the generalizability of the results. This design was selected primarily to avoid comparison to normal brain subjects which could increase the likelihood of uninformative differences. However, this may have also led to a laterality effect within the frontal lobe localized subject pool. Frontal lobe GBM classified pathology had a laterality R:L ratio of 11:1. Having a more laterally balanced frontal GBM group should be an aim for future studies. Similarly, pathology case ratios for frontal, parietal, and temporal lobe localized gliomas were (#GBM cases: #Astrocytoma cases: #Oligodendroglioma cases) 12:5:5, 3:0:2 and 3:2:1, respectively, prohibiting a lobe location and pathology controlled analysis due to small sample size. This analysis should be an aim for future studies given more aggressive tumours (GBM) are expected to have higher degradative effects on surrounding tissue. DSI Studio’s AAL2 atlas was also utilized to parcellate each subject’s brain. However, this atlas is most commonly used for anatomically sound brains. With a presence of a tumour, brain anatomy is distorted. Though, the atlas uses reliable landmark while parcellating the brain and this method was intended to increase consistency and objectivity across subjects, areas near the lesion may be altered/shifted and could impact the results of the connectivity study in terms of where brain region fibres are actually terminating. A registration *R*^2^ value of 0.60 was used as the cut-off for acceptable registration in this study, however, a higher value should be considered in future studies to aid with reliability. Finally, improvement of this study in future projects should include patients with occipital lobe localized gliomas and patient data not restricted to glioma localization near motor/language pathways.

## Conclusion

In this study, we showed preliminary evidence of altered ipsilesional nodal network property and structural connectivity characteristic of frontal, parietal or temporal lobe localized gliomas. This study identified unique sets of distal nodes which demonstrated a change in network efficiency and modularity traits depending on lesion location. This study also highlighted specific ipsilesional connections which showed a significant change in connectivity and the corresponding microstructural degradation, most of which were proximal to lesion location. These results call for further histopathological and molecular studies to better understand the mechanisms underlying these unique anatomical changes. Identifying the structural changes and mechanisms characteristic of lobe localized gliomas will lead to better understanding of underlying symptomology and functional deficits commonly seen in such cases.

## Supplementary material

Supplementary material is available at *Brain Communications* online.

## Supplementary Material

fcaa216_Supplementary_Data

## Abbreviations

AAL2 =: automated anatomic labelling atlas 2
BC =: betweenness centrality
CC =: cluster coefficient
DTI =: diffusion tensor imaging
EC =: eigenvector centrality
FA =: fractional anisotropy
GBM =: glioblastoma
LE =: local efficiency
MD =: mean diffusivity
MNI =: Montreal Neurological Institute
