# Supplementary material for: Retrospective analysis of hemispheric structural network change as a function of location and size of glioma
Source: Brain Commun. 2020 Dec 17;3(1):fcaa216. doi: 10.1093/braincomms/fcaa216 (PMC7811759; doi:10.1093/braincomms/fcaa216)
Supplement: fcaa216_Supplementary_Data [file fcaa216_supplementary_data.docx]

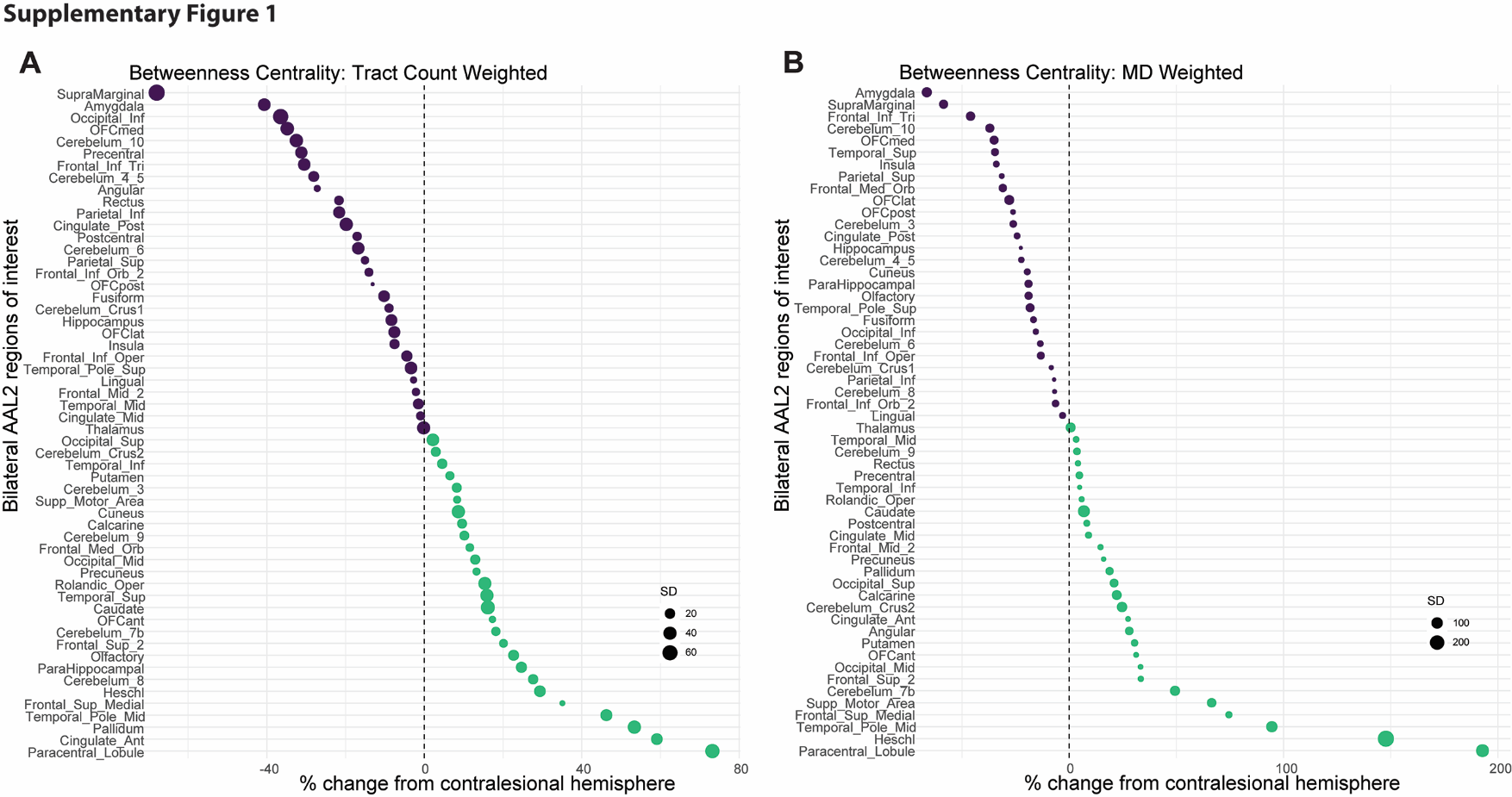


**Supplementary Figure 1**

A.) Percent change in tract count weighted betweenness centrality from the contralesional hemisphere for each AAL2 identified node.

B.) Percent change in MD weighted betweenness centrality from the contralesional hemisphere for each AAL2 identified node.

A list of all anatomical abbreviations is provided in Supplementary Table 1.


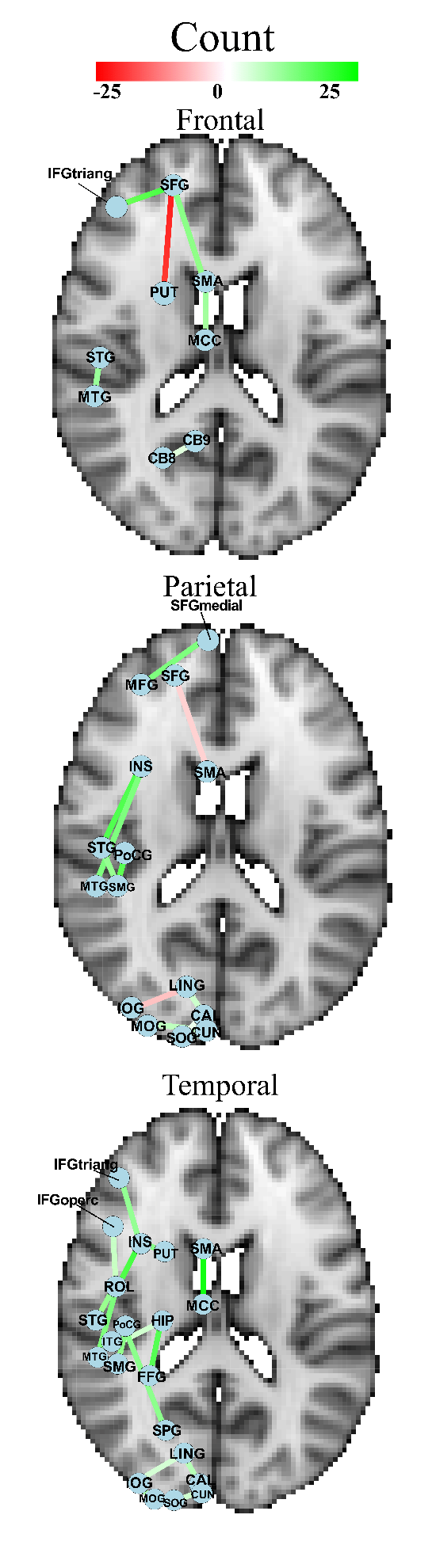


Supplemental Figure 2:

An axial anatomical representation of mean change in end-point tract count of significantly affected connections in frontal, parietal, and temporal lobe localized glioma cases. A list of all anatomical abbreviations is provided in Supplementary Table 1.


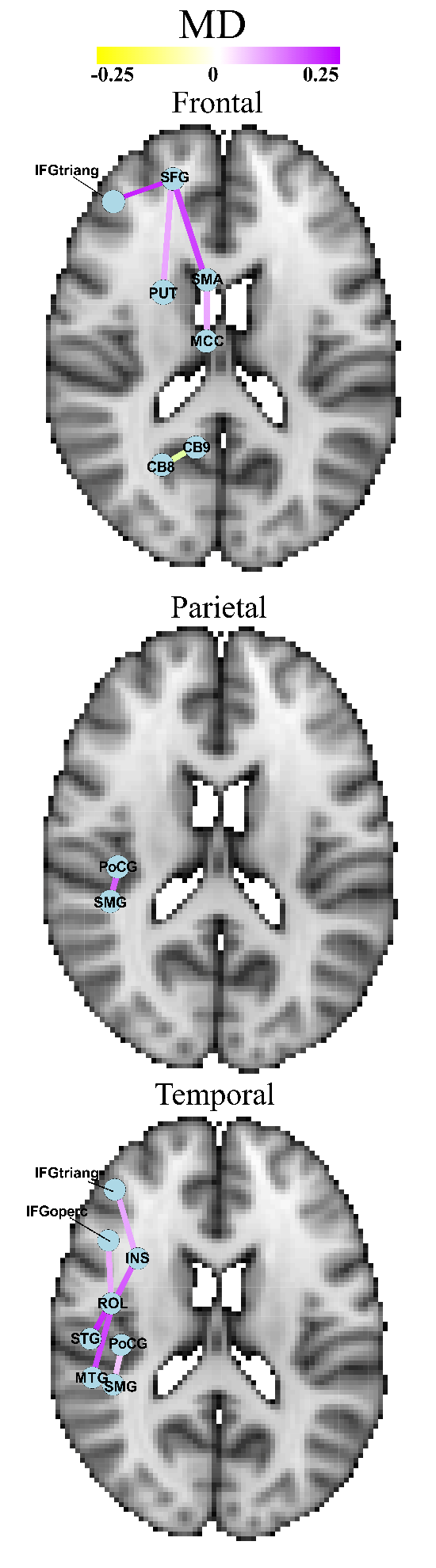


Supplemental Figure 3:

An axial anatomical representation of mean change in MD of significantly affected connections (by change in end-point tract count) in frontal, parietal, and temporal lobe localized glioma cases. A list of all anatomical abbreviations is provided in Supplementary Table 1.

**Supplementary Table 1**

| **POSSIBLE ABBREVIATION** | **ANATOMICAL DESCRIPTION** | **LABEL aal2.nii.gz** |
| --- | --- | --- |
| PreCG | Precentral gyrus | Precentral |
| SFG | Superior frontal gyrus, dorsolateral | Frontal_Sup |
| MFG | Middle frontal gyrus | Frontal_Mid |
| IFGoperc | Inferior frontal gyrus, opercular part | Frontal_Inf_Oper |
| IFGtriang | Inferior frontal gyrus, triangular part | Frontal_Inf_Tri |
| IFGorb | IFG pars orbitalis, | Frontal_Inf_Orb |
| ROL | Rolandic operculum | Rolandic_Oper |
| SMA | Supplementary motor area | Supp_Motor_Area |
| OLF | Olfactory cortex | Olfactory |
| SFGmedial | Superior frontal gyrus, medial | Frontal_Sup_Med |
| PFCventmed | Superior frontal gyrus, medial orbital | Frontal_Med_Orb |
| REC | Gyrus rectus | Rectus |
| OFCmed | Medial orbital gyrus | OFCmed |
| OFCant | Anterior orbital gyrus | OFCant |
| OFCpost | Posterior orbital gyrus | OFCpost |
| OFClat | Lateral orbital gyrus | OFClat |
| INS | Insula | Insula |
| ACC | Anterior cingulate & paracingulate gyri | Cingulate_Ant |
| MCC | Middle cingulate & paracingulate gyri | Cingulate_Mid |
| PCC | Posterior cingulate gyrus | Cingulate_Post |
| HIP | Hippocampus | Hippocampus |
| PHG | Parahippocampal gyrus | ParaHippocampal |
| AMYG | Amygdala | Amygdala |
| CAL | Calcarine fissure and surrounding cortex | Calcarine |
| CUN | Cuneus | Cuneus |
| LING | Lingual gyrus | Lingual |
| SOG | Superior occipital gyrus | Occipital_Sup |
| MOG | Middle occipital gyrus | Occipital_Mid |
| IOG | Inferior occipital gyrus | Occipital_Inf |
| FFG | Fusiform gyrus | Fusiform |
| PoCG | Postcentral gyrus | Postcentral |
| SPG | Superior parietal gyrus | Parietal_Sup |
| IPG | Inferior parietal gyrus, excluding supramarginal and angular gyri | Parietal_Inf |
| SMG | SupraMarginal gyrus | SupraMarginal |
| ANG | Angular gyrus | Angular |
| PCUN | Precuneus | Precuneus |
| PCL | Paracentral lobule | Paracentral_Lobule |
| CAU | Caudate nucleus | Caudate |
| PUT | Lenticular nucleus, Putamen | Putamen |
| PAL | Lenticular nucleus, Pallidum | Pallidum |
| THA | Thalamus | Thalamus |
| HES | Heschl’s gyrus | Heschl |
| STG | Superior temporal gyrus | Temporal_Sup |
| TPOsup | Temporal pole: superior temporal gyrus | Temporal_Pole_Sup |
| MTG | Middle temporal gyrus | Temporal_Mid |
| TPOmid | Temporal pole: middle temporal gyrus | Temporal_Pole_Mid |
| ITG | Inferior temporal gyrus | Temporal_Inf |
| CERCRU1 | Crus I of cerebellar hemisphere | Cerebellum_Crus1 |
| CERCRU2 | Crus II of cerebellar hemisphere | Cerebellum_Crus2 |
| CER3 | Lobule III of cerebellar hemisphere | Cerebellum_3 |
| CER4_5 | Lobule IV, V of cerebellar hemisphere | Cerebellum_4_5 |
| CER6 | Lobule VI of cerebellar hemisphere | Cerebellum_6 |
| CER7b | Lobule VIIB of cerebellar hemisphere | Cerebellum_7b |
| CER8 | Lobule VIII of cerebellar hemisphere | Cerebellum_8 |
| CER9 | Lobule IX of cerebellar hemisphere | Cerebellum_9 |
| CER10 | Lobule X of cerebellar hemisphere | Cerebellum_10 |
| VER1_2 | Lobule I, II of vermis | Vermis_1_2 |
| VER3 | Lobule III of vermis | Vermis_3 |
| VER4_5 | Lobule IV, V of vermis | Vermis_4_5 |
| VER6 | Lobule VI of vermis | Vermis_6 |
| VER7 | Lobule VII of vermis | Vermis_7 |
| VER8 | Lobule VIII of vermis | Vermis_8 |
| VER9 | Lobule IX of vermis | Vermis_9 |
| VER10 | Lobule X of vermis | Vermis_10 |

Supplementary Table 1:

List of all AAL2 Atlas abbreviations and anatomical descriptions. Table was adapted from Rolls et al. (Rolls et al., 2020).
